# Editorial: Hypoxia, oxidative stress, and endocrine cancers

**DOI:** 10.3389/fendo.2023.1268268

**Published:** 2023-10-16

**Authors:** Bertrand Duvillié, Ralf Jockers

**Affiliations:** ^1^ Department of Signaling, Radiobiology and Cancer, Institut Curie, Orsay, France; ^2^ Inserm U1021, Centre Universitaire, Orsay, France; ^3^ CNRS UMR 3347, Centre Universitaire, Orsay, France; ^4^ Université Paris-Saclay, Orsay, France; ^5^ PSL Research University, Paris, France; ^6^ Université Paris Cité, Institut Cochin, Inserm, CNRS, Paris, France

**Keywords:** hypoxia, oxidative stress, endocrine tumors, radiotherapy, HIF - 1α

The Research Topic “*hypoxia, oxidative stress and endocrine cancers*” aims to present innovative strategies to investigate the role of hypoxia and oxidative stress in endocrine cancers. Such analysis of the hypoxic environment will allow an improved understanding of the contribution of the cellular and molecular signaling pathways associated with tumor initiation, progression, and metastasis, and the subsequent knowledge will help to improve cancer treatments such as chemotherapy and radiotherapy.

Oxygen pressure (pO_2_) is involved in many important biological functions, including metabolism, proliferation, angiogenesis, and apoptosis, while dysregulation of oxygen metabolism has been implicated in a number of cancers. Research has indicated that the pO_2_ varies widely between different tissues, with many being hypoxic, with a concentration range from 6 to 34 mmHg (1% to 5% O_2_) (1). Indeed, in the atmosphere and upper airways, the oxygen level is 160 mmHg (21% O_2_) and 150 mmHg (19.7% O_2_), respectively (2). The pO_2_ of the arterial blood is generally 95 mmHg (12.5% O_2_), decreasing to 40 mmHg (5.2% O_2_) in venous blood. Conversely, in the mouse thymus, the oxygen value was estimated at only 7.6 mmHg (1% O_2_) (3). Semenza et al. indicated that the hypoxia-inducible factors (HIFs) play a central role in the signaling pathways involved in cancer progression. The HIF1α protein is generally expressed in tissues when pO_2_ is below 34 mmHg (5% O_2_). HIF transcription factors control the expression of thousands of genes, regulating crucial mechanisms, including angiogenesis, cancer stem cell specification, cell motility, epithelia-mesenchymal transition, extra-cellular matrix remodeling, glucose and lipid metabolism, immune evasion, invasion, and metastasis (4). The level of HIF protein expression is tightly regulated by both oxygen-dependent and independent signals to ensure a functional equilibrium.

An example of the concentration effect of HIF1α can be seen in the study of pancreatic beta cell function (5). Indeed, HIF1α is present at very low levels under physiological conditions in both mouse and human beta-cells. A beta-cell specific HIF1α disruption in mice leads to glucose intolerance and beta-cell dysfunction (6). Interestingly, the gain of function of HIF1α, induced by the deletion of the tumor suppressor VHL, leads to defective beta-cell differentiation (7) and altered beta-cell function (8–10).

This phenotype is similar to that caused by the loss of HIF1α function. Thus, this counterintuitive observation highlights the importance of HIF1α dosage for endocrine function. Moreover, patients with a mutation in *VHL* develop Von Hippel Lindau disease, with some patients developing cystic serous adenoma, and/or pancreatic neuroendocrine tumors (11).

In this Research Topic, Watts et al. present an overview of the recent finding on the central role of the HIF axis and its inhibitors, the Prolyl Hydroxylase Domain proteins (PHDs), in endocrine tumors. The relationship between oxygen tension and reactive oxygen species (ROS) has previously been established (12) and Cui et al. identified eleven mitochondrial genes related to oxidative stress that are associated with the progression of pancreatic cancer and pancreatic neuroendocrine tumors.

Women’s cancers are also frequently concerned by endocrine tumors and their hypoxic microenvironment. In a recent study, Pereira et al. presented an overview on the role of hypoxia in ovarian cancer, and its effects on the immune environment. Importantly, they analyzed the impact on chemoresistance and discussed the future possibilities of personalized therapies. Chen et al. further established a model based on hypoxia-related gene expression to predict prognosis in endometrial cancer, another female-specific tumor. This model is based on a genetic signature of 4 genes: *ANXA2, AKAP12, NR3C1*, and *GPI*. Finally, Quinting et al. analyzed the role of myoglobin, an oxygen-binding protein that exhibits a scavenging capacity for reactive oxygen and nitrogen species, in breast cancer. Their results strongly suggest that myoglobin supports the survival of breast cancer cells due to its scavenging properties.

The role of oxidative stress in cancer has also been extended to other types of tumors. For example, Dong et al. showed that a novel molecular classification incorporating oxidative stress and metabolism-related genes could be used for prognosis prediction and personalized medicine in stomach adenocarcinoma.

Finally, in the last part of this Research Topic, the application of hypoxia studies to cancer therapy is proposed. Indeed, it was shown that oxygen tension can considerably influence radiotherapy efficiency, with hypoxia being one of the most important causes of radioresistance. Moreover, hypoxia is associated with a poor prognosis after radiotherapy. Rakotomalala et al. analyzed the different mechanisms by which hypoxia may influence the efficacy of radiotherapy in solid tumors. In particular, they detail the 6 parameters considered in the field of radiotherapy, known as the “6Rs of radiation biology”: Radiosensitivity, Repair, Repopulation, Redistribution, Reoxygenation, and Reactivation of anti-tumor immune response. This review presents innovative data concerning the effects of hypoxia during radiotherapy, and describes the results of recent clinical trials. To return to the main focus of this Research Topic, endocrine tumors, the authors analyze these effects in the case of anaplastic thyroid carcinoma.

Overall, this Research Topic presents new concepts on the role of hypoxia and oxidative stress in endocrine cancer, while also providing new tools and directions to classify the patients and to delineate the molecular mechanisms for a future optimization of the treatments and the development of a personalized medicine.

## Author contributions

BD: Conceptualization, Investigation, Validation, Writing – original draft, Writing – review & editing. RJ: Validation, Writing – original draft, Writing – review & editing.
